# The Impact of Celiprolol in Vascular Ehlers–Danlos Syndrome: A Systematic Review of Current Evidence

**DOI:** 10.3390/medsci13020074

**Published:** 2025-06-09

**Authors:** Pandit Bagus Tri Saputra, Wynne Widiarti, Paulus Parholong Siahaan, Rendra Mahardhika Putra, Johanes Nugroho Eko Putranto, Raden Mohammad Budiarto, Nadya Luthfah, Chaq El Chaq Zamzam Multazam, Mario D’Oria, Firas Farisi Alkaff

**Affiliations:** 1 Department of Cardiology and Vascular Medicine, Dr. Soetomo General Academic Hospital, Surabaya 60286, Indonesia; panditbagusts@gmail.com (P.B.T.S.); j.nugroho.eko@fk.unair.ac.id (J.N.E.P.); raden-m-b@fk.unair.ac.id (R.M.B.); nadya.luthfah@fk.unair.ac.id (N.L.); 2 Department of Cardiology and Vascular Medicine, Faculty of Medicine, Universitas Airlangga, Surabaya 60132, Indonesia; 3 Faculty of Medicine, Universitas Airlangga, Surabaya 60132, Indonesia; wynnewidiartii@gmail.com (W.W.); paulusparholongsiahaan@gmail.com (P.P.S.); 4 National Heart and Lung Institute, Imperial College London, London SW3 6LY, UK; zamzam.multazam23@imperial.ac.uk; 5 Division of Vascular and Endovascular Surgery, Department of Clinical Surgical and Health Sciences, University of Trieste, 34127 Trieste, Italy; 6 Division of Nephrology, Department of Internal Medicine, University Medical Center Groningen, 9713 GZ Groningen, The Netherlands; f.f.alkaff@umcg.nl; 7 Division of Pharmacology and Therapy, Department of Anatomy, Histology, and Pharmacology, Faculty of Medicine, Universitas Airlangga, Surabaya 60132, Indonesia

**Keywords:** Ehlers–Danlos syndrome, beta-blockers, celiprolol, arterial rupture, collagen disorders, vascular disease

## Abstract

Objectives: Ehlers–Danlos syndrome (EDS) is a group of connective tissue disorders characterized by mutations affecting collagen and extracellular matrix proteins. Vascular EDS (vEDS) stands out for its severe prognosis due to the heightened risk of arterial and organ rupture which significantly increase mortality rates. Limited strategies for treating vEDS are prompting exploration for alternatives such as celiprolol, a cardioselective beta-blocker with potential to reduce vascular stress and improve collagen integrity. This review aims to evaluate current evidence on the impact of celiprolol in managing vEDS. Methods: A comprehensive literature search was conducted across scientific databases for studies comparing celiprolol with placebo or other treatments, focusing on relevant outcomes. Results: A total of 323 participants were included across studies published from 2010 to 2023, primarily conducted in European settings. Celiprolol administration, starting at 100 mg daily and titrated up to 400 mg, significantly reduced the incidence of major vascular events such as arterial dissections and ruptures. Most studies reported improved survival rates and fewer hospitalizations due to acute arterial events. Variations in treatment response and side effects such as dizziness and hypotension were noted across studies, occasionally leading to treatment. Conclusions: Celiprolol appears to be a promising treatment for reducing vascular events in vEDS patients, potentially improving quality of life and mitigating the substantial morbidity and mortality associated with vEDS. Future research should focus on refining treatment protocols, exploring mechanisms of action, and establishing comprehensive clinical guidelines to optimize patient outcomes.

## 1. Introduction

Ehlers–Danlos syndrome (EDS) encompasses a group of connective tissue disorders caused by mutations affecting collagen and extracellular matrix protein synthesis and processing. According to the 2017 International EDS Consortium, EDS can be classified into 13 subtypes [1]. Vascular Ehlers–Danlos syndrome (vEDS) is the most severe subtype, with a median survival of 40–50 years. vEDS arises from heterozygous mutations in the *COL3A1* gene and in some cases the *COL1A1* gene. Mutations in the *COL3A1* gene specifically result in defects in the pro alpha-1(III) chain of type III collagen [2,3]. These genetic defects compromise connective tissue integrity, leading to a dramatically increased risk of vascular and hollow organ rupture and significantly affecting morbidity and mortality. By age 40, approximately 90% of individuals with vEDS will have experienced a major complication, often beginning as early as their 20s [4]. It is inherited in an autosomal dominant manner and also has a distinct genetic and phenotypic profile that requires specialized clinical approaches.

Celiprolol, a dual-action beta-blocker with beta-1 antagonism and beta-2 agonist vasodilatory properties, has been shown to reduce mechanical stress on collagen fibers in the vascular wall, thus increasing their durability [5]. Recent studies highlight the impact of celiprolol on transforming growth factor-beta (TGF-beta) expression and its beta-adrenergic activity among other mechanisms [6]. A 2010 Randomized Controlled Trial (RCT), Beta-Blocker in Ehlers–Danlos Syndrome Treatment (BBEST), demonstrated celiprolol’s efficacy in reducing arterial rupture incidence by up to 30% [7]. Given the potential of celiprolol to mitigate complications associated with vEDS pathophysiology, this review aims to summarize the current evidence regarding its impact on patients with vEDS.

## 2. Methods

This systematic review followed the Preferred Reporting Items for Systematic Reviews and Meta-Analyses (PRISMA) 2020 guideline and the Cochrane Handbook for Systematic Review of Interventions and was registered in the PROSPERO database under the registration number of CRD42024553226 [8] (Supplementary Table S1).

### 2.1. Data Search Strategy

A systematic literature search was conducted until 13 July 2024, searching through several databases including Pubmed, ScienceDirect, Scopus, Springer, Web of Science, and Proquest, using keywords and Boolean operators of ((Celiprolol) AND (Ehlers-Danlos Syndrome)). The search terms were applied to the titles, abstracts, and the entire text of the publications. Manual searching was also performed to provide larger coverage of gray literature. Two authors (WW and PPS) independently screened for the search results, with discrepancies resolved by the third author (PBTS).

### 2.2. Eligibility Criteria

This systematic review included observational studies and clinical trials with the following inclusion criteria: (1) patients with vEDS aged older than 17 years; (2) studies comparing celiprolol with placebo or other treatments; (3) reporting any outcomes of interest; (4) written in English. This systematic review excluded case reports, case series, editorial letters, animal studies, and review articles.

### 2.3. Data Extraction and Quality Assessment

The authors extracted any relevant data from the included studies, including the authors’ ID (Name and published year); study period; follow-up period; study design; study location; sample size; age; BMI; comorbidities including diabetes mellitus, hypertension, dyslipidemia, previous history of arterial, gastrointestinal, and pulmonary events; variants of vEDS; treatment characteristics of celiprolol such as target dose; duration to reach target dose; side effects and impact of treatment including new vascular events (any arterial rupture, thrombosis, dissection, or aneurysm); gastrointestinal (bowel bleeding or rupture) and pulmonary events (pneumo- or hemothorax); and mortality that occurred after treatment initiation and during the follow-up period.

### 2.4. Quality Assessment

The quality of included studies was evaluated using the Newcastle–Ottawa Scale (NOS) quality assessment tool, obtaining points from assessing three different aspects: selection (4 marks), comparability (2 marks), and outcomes (3 marks) [9]. Two authors (WW and PPS) independently carried out the bias assessment, and differences in the final results were resolved by the third author (PBTS). The detailed results of the quality assessment are presented in Supplementary Table S2.

### 2.5. Statistical Analysis

A meta-analysis was not feasible due to the diverse range of data included in this systematic review. Therefore, the available evidence was synthesized and examined using a qualitative approach.

## 3. Results

### 3.1. Study Selection and Quality Assessment

**Figure 1:**
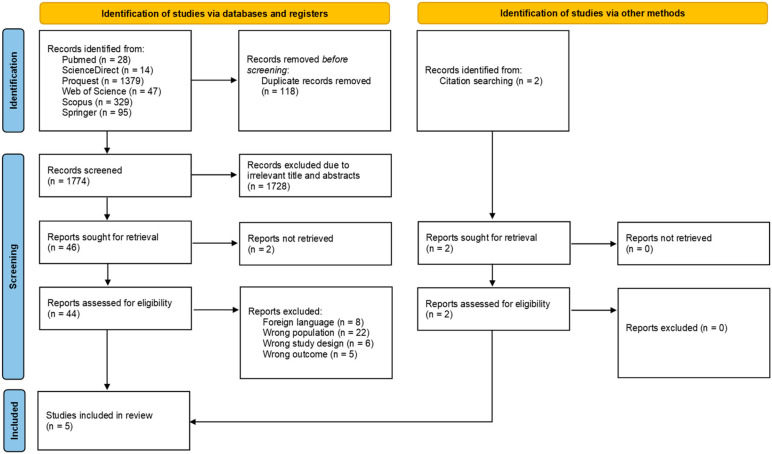
PRISMA flow diagram of the study selection process.

A systematic search was conducted across six databases and yielded a total of 1892 studies. After electronically detecting and excluding 118 duplicates, the screening process advanced to reviewing titles and abstracts, which narrowed down the studies for further inclusion. Following an assessment of 44 studies, 5 studies (published in 2010–2023) were included in this systematic review [6,7,10,11,12]. Details regarding study selection and reason of exclusion are presented in the PRISMA flow diagram (Figure 1).

### 3.2. Baseline Characteristics

A total of 323 subjects with an average age from 30 to 78 years were included in this systematic review. The percentage of male subjects ranged from 32 to 43%. Most studies were conducted in European countries including Sweden [6], Italy [10], France [7,11,12], and Belgium [7]. The baseline characteristics and comorbidities are summarized in the Supplementary Tables S3 and S4.

### 3.3. vEDS Type and Clinical Manifestation

Despite using different criteria, all included subjects were diagnosed with vEDS based on clinical and molecular criteria [6,7,10,11,12]. A study by Baderkhan et al. (2021) [6] classified their subjects based on the guideline of American College of Medical Genetics (ACMG) (2021) [6], while Frank et al. (2019) [11] classified their subjects based on molecular analysis namely glycine missense mutations, splice-site variants, and those leading to haploinsufficiency. The most common variant treated and included in this particular study was the glycine missense mutation. Similarly, Buso et al. (2024) [10] and Frank et al., 2019 [11] found that the glycine missense mutation was the most prevalent variant. Frank et al., 2019 [11] specifically documented that glycine substitutions were associated with earlier and more severe vascular complications, including arterial dissections and ruptures, compared to splice-site mutations or null alleles, which were linked to milder phenotypes. Ong et al. (2010) [7] evaluated subjects based on clinical and molecular features adapted from the Villefranche criteria, consisting of three major and ten minor criteria, of which most patients were presented with three major criteria. Hoang et al. (2014) [12] did not mention any molecular classification used in the study.

### 3.4. Treatment Characteristics and Adverse Effects

All studies reported that the celiprolol treatment started with an initial dose of 100 mg that was gradually titrated up to a target dose of 400 mg per day [6,7,10,11,12]. The uptitration process in most studies was conducted incrementally every 6 months in the study by Baderkhan et al. (2021) [6] and Ong et al. (2010) [7]. In contrast, Frank et al. (2019) [11] increased the dose by 100 mg each month over a 3-month period [11]. A total of 135 out of 189 participants (71%) across the pooled studies tolerated the target dose of celiprolol. The total number of participants (n = 189) is derived from the combined sample sizes of the studies listed in Supplementary Table S3. A study by Baderkhan et al. (2021) [6] reported that 65% of all participants reached the target dose. Details regarding celiprolol treatment were presented in Supplementary Table S5.

In the study by Baderkhan et al., 2021 [6], there were fourteen patients experiencing side effects, with six (15%) facing severe reactions such as dizziness (n = 2; 5%), hypotension (n = 1; 2.5%), imbalance (n = 1; 2.5%), deterioration of asthma (n = 1; 2.5%), and headache (n = 1; 2.5%) that hindered achieving the desired target dose. The most frequent side effects included dizziness (n = 5; 35.7%), fatigue (n = 4; 28.57%); headaches (n = 2; 14.29%), and nausea (n = 2; 14.29%). The majority of these side effects were temporary and subsided after the medication dose was lowered. In addition, four patients terminated celiprolol due to side effects and one for alleged economic reasons [6].

On the other hand, Buso et al. (2024) [10] only reported a case (3.85%) of fatigue attributed to intolerance at a higher dosage, where the patient reached a maximum dose of 200 mg of celiprolol. Two patients (8%) died during drug titration. In total, 80% of patients reach the recommended dose [10]. In the study by Frank et al. (2019), 62.5% of the total subjects were able to tolerate the maximum dose regimen [11], while 5 (3.5%) patients experienced fatigue and required a dose reduction. This study also observed that there were no serious adverse events related to the drug reported [11]. Ong et al. (2010) [7] documented two (8.70%) cases of mild fatigue, which was associated with a dosage increase to 300 mg. In total, 88% of participants reached the maximum celiprolol dose. No cases of clinical hypotension or bradycardia were documented in this study. However, two patients had a dose reduction due to fatigue, and one patient also immediately stopped the regimen after receiving the initial dose at 100 mg due to severe fatigue [7]. Details regarding the side effects of celiprolol are summarized in Supplementary Table S6.

### 3.5. The Impact of Celiprolol Treatments

#### 3.5.1. Clinical Parameters

Patients treated with celiprolol showed notable changes in clinical parameters including blood pressure [6,7,12], pulse pressure [6,7,12], and pulse rate [6]. In terms of blood pressure, a study by Hoang et al. (2014) [12] reported an increase in SBP by 0.79 mmHg per year and central SBP by 0.89 mmHg per year [12]. Similarly, Ong et al. (2010) [7] also reported an increasing brachial systolic pressure and pulse pressure after celiprolol treatment [7]. In contrast, Baderkhan et al. (2021) [6] reported a decrease in systolic (127 ± 14.5 mmHg vs. 120 ± 13.3 mmHg; *p* < 0.001) and diastolic blood pressure (82 ± 14.2 vs. 75 ± 11.5 mmHg; *p* < 0.001) [6]. In terms of central pulse pressure, Hoang et al. (2014) [11] reported an increase in central pulse pressure by 1.24 mmHg per year, with no changes in heart rate [11]. However, Baderkhan et al. (2021) [6] reported a decrease in mean pulse pressure (45 ± 10.8 mmHg vs. 44 ± 14.2 mmHg; *p* = 0.021) and pulse rate (76 ± 5.7 beats/minute (bpm) vs. 70 ± 8.1 bpm; *p* = 0.14) [6]. In addition, Buso et al. (2024) [10] observed no significant changes over time in blood pressure and pulse pressure after celiprolol treatment. In addition, Ong et al. (2010) [7] also reported that celiprolol treatment in vEDS increased arterial stiffness, as evidenced by reduced carotid distensibility and increased Young’s elastic modulus, alongside elevated pulse wave velocity, suggesting enhanced arterial wall strength via beta2-adrenergic stimulation and TGF-beta-mediated collagen synthesis. Hoang et al. (2014) [12] corroborated these findings in a 5-year follow-up, showing increased Young’s elastic modulus (+29.92 kPa/y, *p* < 0.001), reduced distensibility (−0.003 kPa^−1^/y, *p* < 0.001), and growth in carotid intima-media thickness (+4.4 μm/y, *p* < 0.001), with similar trends observed in untreated patients, indicating these changes may reflect aging rather than celiprolol-specific effects [12].

#### 3.5.2. Vascular Events

Regarding the occurrence of new vascular events, after the mean follow-up duration of 72 ± 41 months, a single-arm cohort by Buso et al. (2024) [10] reported that 70% (n = 14/20) of patients experienced one or more major vEDS-related event during their follow-up period, with a total of 12 vascular events and 2 gastro-intestinal events. The reported symptomatic vascular events included two cases of type B aortic dissection (n = 2/18; 11.1%), three cases of renal artery thrombosis (n = 3/18; 16.7%) or dissection leading to renal infarction, two cases of hepatic artery rupture (n = 2/18; 11.1%), one case of splenic artery rupture (n = 1/18; 5.6%), and also three cases of iliac artery thrombosis or dissection (n = 3/18; 16.7%). Notably, 10 of the 18 events (55.6%) occurred after patients reached the recommended dose of celiprolol, which is 400 mg daily [10].

Similar outcomes were obtained by a random clinical trial by Ong et al. (2010) [7] where in the celiprolol group, only 20% (5 out of 25) experienced vascular events, versus 50% (14 out of 28) in the control group, translating to a 64% risk reduction with celiprolol treatment. In addition, this study also suggests that low diastolic (<62 mmHg) or high pulse pressures (>50 mmHg) were indicators of negative outcomes for the occurrence of vascular events [7]. Baderkhan et al. (2021) [6] specifically reported that five major vascular events occurred during the follow-up period, resulting in an annual risk of a major vascular event of 4.7%. Four of which were fatal and caused mortality, including ruptured ascending aorta, aortic rupture after type B dissection, ruptured cerebral aneurysm, and ruptured pulmonary artery. One event involved bleeding from a branch of the internal iliac artery but was successfully treated with endovascular coiling. Among these five patients, three of which (60%) shared common characteristics, the baseline pulse pressure was >50 mmHg [6].

Conversely, an observational study conducted by Frank et al. (2019) [11] documented no significant difference in the overall incidence of arterial events or aggregate survival rates between treatment and control groups during the initial follow-up period. However, during a median follow-up duration of 5.3 years, this study documented a significant reduction in hospitalizations for acute arterial events after the initiation of celiprolol, from 41 (17.7%) to 39 (11.2%). This change is statistically significant, with an odds ratio of 1.7 (*p* = 0.0257), suggesting a positive effect of celiprolol on reducing the severity of new arterial events. Moreover, this study also emphasized the importance of achieving and maintaining the recommended dose to maximize therapeutic benefits, with the greatest benefit observed in patients taking 400 mg daily [11].

#### 3.5.3. Mortality

Baderkhan et al. (2021) [6] reported four cases of mortality during and after the follow-up. The first case is a 59-year-old woman who died due to cardiac tamponade, secondary to type A aortic dissection. This patient had only received 100 mg celiprolol daily for two months. The next case is subarachnoid bleeding due to ruptured cerebral aneurysm in a 73-year-old man, who had received 400 mg celiprolol daily for three years. The third mortality case is a 44-year-old man who died due to an ascending aortic rupture. This patient had been treated with 400 mg celiprolol daily for five years. Similarly, the fourth case is a 62-year-old man who died due to pulmonary artery rupture. This patient had been receiving 400 mg celiprolol daily for 4.5 years. All of these patients had high baseline pulse pressure (>50 mmHg), but only two of which remained high even after celiprolol initiation [6]. In addition, Buso et al. (2024) [10] also reported three mortality cases due to vascular events, especially splenic artery rupture. In these cases, all of them received celiprolol treatment: a 15-year-old boy and a 37-year-old woman due to type B aortic dissection and hepatic artery rupture during splenectomy for spontaneous spleen rupture and a 42-year-old man from splenic artery rupture. Unfortunately, this study did not report the specific dose and duration of celiprolol therapy [10].

However, a study by Frank et al. (2019) [11] revealed detailed analysis on the survival curve that indicated patients not treated with celiprolol experienced significantly lower survival rates compared to those who received the treatment, particularly after 11.1 years of follow-up (with celiprolol 19.3% (95% CI: 6.4% to 32.2%) vs. without celiprolol 51.5% (95% CI: 22.6% to 80.3%); *p* < 0.001). Further analysis revealed that among those treated with celiprolol, patients receiving 400 mg/day (n = 83/132; 62.9%) showed a significantly higher survival rate compared to those receiving between 100 and 300 mg/day (n = 27/132; 20.5%). This study concluded that the greatest protective effect was seen at a dosage of 400 mg/day compared to doses less than 400 mg/day (*p* = 0.003) [11]. Another study by Ong et al. (2010) [7] reported one death among patients treated with celiprolol (n = 1/25; 4%) accompanied with acute chest pain radiating to the right arm. Unfortunately, the cause of death of this 19-year-old man remained unclear. On the other hand, in the control group, there were three deaths (n = 3/28; 10.7%). In these cases, one case was due to iliac artery rupture, one case was due to aortic dissection, and one case remained unclear but was accompanied with acute lumbar pain. This study also did not report the specific dose and duration of celiprolol therapy [7]. Table 1 summarizes the dosing strategies, vascular event incidence, adverse effects, and mortality outcomes from the five included studies, highlighting inter-study variations and common trends.

## 4. Discussion

This systematic review highlights the role of celiprolol in reducing vascular events in vEDS patients. Across five cohort studies, celiprolol treatment was also reported to enhance survival rates, particularly when dosages reached 400 mg daily [6,7,10,11,12]. Despite some reported side effects such as dizziness and fatigue, the majority of patients can tolerate the maximum dose. Notably, the included studies emphasized the importance of a personalized dosing regimen to maximize patient outcomes and minimize adverse effects. These findings are clinically relevant considering the underlying pathology of vEDS, which stems from mutations in the *COL3A1* gene encoding type III collagen. This type of collagen is particularly important for maintaining physiological functions such as cell differentiation, wound healing, angiogenesis, and structural support of smooth muscle. Consequently, vEDS leads to compromised tissue integrity across various organ systems and increased wall stress in the vascular system, raising the risk of vessel rupture or dissection [13]. With the fragility in their blood vessels, patients with vEDS carry significant morbidity and mortality. However, there are no established treatments for vEDS, resulting in patients being managed expectantly [14].

Patients with vEDS are often empirically treated with beta-blocker or renin–angiotensin–aldosterone blockers that were proven to be beneficial in Marfan syndrome. Unfortunately, there are significant differences in the pathophysiology of Marfan syndrome and vEDS [15,16]. Marfan syndrome is characterized by decreased aortic distensibility, increased stiffness index, and increased pulse wave velocity due to fibrillin-1 deficiency and abnormal elastin synthesis. This leads to aortic stiffening and dilation. In contrast, vEDS is marked by blood vessel fragility that affects the entire arterial tree, skin, and intestines, resulting in increased wall stress and a high risk of vessel rupture or dissection [17]. Additionally, vEDS also involves a decrease in intima-media thickness and increased mechanical stress on fragile tissues, features that were not observed in Marfan syndrome, where aortic stiffening and dilation are predominant [18]. Hence, Ong et al. (2010) [7] initiated the use of celiprolol treatment in vEDS patients. It is hypothesized that celiprolol, a cardio-selective beta-blocker with beta-2 agonist vasodilatory properties, would reduce central blood pressure and mechanical load on arterial collagen fibers through lowering heart rate and blood pressure, thus preventing dissection and rupture [7]. Subsequent preclinical studies in mouse models of vEDS have also provided important mechanistic support for this clinical approach [19,20]. These studies demonstrated that celiprolol significantly improves the biomechanical integrity of the thoracic aorta, particularly when compared to losartan, which showed no beneficial effect in the same models. This reinforces celiprolol’s role in protecting against arterial rupture, potentially through mechanisms beyond its antihypertensive effects [19,20].

Currently, there is limited evidence on the clinical impact of celiprolol in managing vEDS. The effect of reduction in blood pressure and pulse pressure was primarily reported in the study by Baderkhan et al. (2021) [6]. The changes in clinical parameters are likely due to its pharmacodynamic properties, including the blockade of beta-1 adrenoreceptors and decreased cardiac contractility in both the sinoatrial node and myocardium [21,22]. Jeunemaitre et al. (2025) also explored celiprolol in combination with irbesartan, demonstrating that combination therapy may yield additional protective effects beyond monotherapy, although further validation is needed [22]. Additionally, its beta-3-adrenoreceptor agonistic activity and stimulation of nitric oxide release result in vasodilation and consequently also lowered blood pressure and the pulse rate. This effect potentially reduces vascular oxidative stress and benefits the extracellular matrix. However, the exact mechanism through which celiprolol strengthens the aortic wall remains unclear. A single dose of 400 mg was proven to lower standing DBP up to 10%, without adversely affecting cardiac function, but the BP lowering effects were not applicable in normotensive patients [23]. Thus, the protective effect of celiprolol may not be due to blood pressure reduction. Instead, celiprolol is assumed to provide better stability in hemodynamic conditions and reduce arterial fragility by preventing excessive blood pressure and heart rate peaks during stress and exercise [24,25].

Elevated TGF-beta levels have been observed in vEDS patients, potentially indicating a physiological response to impaired collagen synthesis. This overexpression may contribute to the fragility of the vascular system by altering the balance between extracellular matrix production and degradation [5,12,13]. Additionally, TGF-beta signaling pathways have been implicated in other connective tissue disorders, such as Marfan syndrome and Loeys–Dietz syndrome, suggesting a broader relevance of TGF-beta dysregulation in connective tissue disease pathology. Through the beta-2 stimulation, celiprolol is assumed to exert its protective effects by stabilizing TGF-beta signaling, thus enhancing collagen synthesis and improving the mechanical integrity of blood vessels [26,27]. This theory is supported by evidence of reduced matrix turnover due to suppressed TGF-beta expression, increasing vessel wall elasticity. Reducing the incidence of vascular ruptures or dissections may lower morbidity and mortality rates in vEDS patients, as it addresses the most common cause of mortality and morbidity in this population [28]. While losartan effectively reduces the progression of aortic aneurysm in Marfan syndrome by blocking *AGTR1* and inhibiting TGF-beta signaling, it has been proven that losartan does not improve the biomechanical integrity of the thoracic aorta in the vEDS mouse model [17,18]. Hence, further research is needed to investigate the precise mechanisms by which TGF-beta influences vEDS progression and to explore therapeutic interventions targeting this pathway.

Given the promising results of celiprolol in reducing vascular events in vEDS patients, it is recommended that clinicians consider celiprolol as a treatment option for managing this condition. Patients should start on a lower dose (100 mg) and gradually increase to a maximum of 400 mg [6,7,10,11,12], monitoring for side effects such as dizziness and hypotension. Regular follow-ups and imaging should be conducted to assess vascular health and adjust the treatment regimen as necessary. Additionally, clinicians should educate patients about the potential benefits and risks of celiprolol therapy, emphasizing the importance of adherence to the treatment plan and regular monitoring.

This study has several strengths and limitations. To date, this systematic review is the first to comprehensively investigate the current evidence on the impact of celiprolol treatment in vEDS patients, focusing particularly on vascular events. However, the existing literature is limited. Additionally, certain studies also lacked comprehensive data, especially regarding sample clinical characteristics and the long-term clinical benefits of celiprolol. Furthermore, one of the included studies, Hoang et al. (2014) [12], is a conference abstract and shares several authors with Frank et al. (2019) [11]. While it was included to broaden the scope of evidence on celiprolol use in vEDS, the limited methodological detail in the abstract makes it difficult to determine whether the patient populations overlap. This potential duplication could affect the independence of reported outcomes, and we have noted this limitation to maintain transparency in the interpretation of results. We have also identified a notable gap in the literature concerning the use of celiprolol in surgical contexts, reflecting a broader lack of clinical evidence in this area. Moreover, neither the latest European Society for Vascular Surgery (ESVS) 2024 Clinical Practice Guidelines on the Management of Abdominal Aorto-Iliac Artery Aneurysms [29] nor the most recent EACTS/STS 2024 guidelines [30] provide any specific recommendations or dosing strategies for celiprolol in the postoperative setting, underscoring the need for further research in this domain. Given these constraints, further research is essential to gather more robust evidence and establish precise clinical guidelines for the use of celiprolol in vEDS patients. Future studies should address these gaps by conducting large-scale randomized controlled trials and long-term observational studies to better understand celiprolol’s efficacy, safety, and mechanisms in vEDS. Additionally, research should focus on optimizing dosing strategies, exploring mechanisms of action, and identifying effective combination therapies, while ongoing studies aim to refine clinical guidelines and enhance patient outcomes and quality of life. Notably, a prospective, double-blind, placebo-controlled trial (ClinicalTrials.gov Identifier: NCT05432466) is currently underway and is expected to provide more robust data in the future.

## 5. Conclusions

This systematic review underscores the potential benefits of celiprolol in managing vEDS. Celiprolol, as a cardioselective beta-blocker, has demonstrated efficacy in reducing vascular events and mortality, primarily by lowering blood pressure and pulse rate. Beyond its hemodynamic effects, evidence suggests celiprolol may enhance arterial stability and reduce the risk of arterial rupture during physical or emotional stress. While generally well tolerated, with manageable side effects such as dizziness and fatigue, the protective effects of celiprolol appear to extend beyond blood pressure reduction. Celiprolol should be considered as a therapeutic option for vEDS. Further research is essential to fully elucidate the mechanisms by which celiprolol influences vEDS progression and to develop treatment guidelines for improving patient outcomes.

## Figures and Tables

**Table 1:** Summary of Study Characteristics and Outcomes.

Study	Country	Study Design	Follow-up Duration	Sample Size	Celiprolol Dose and Titration	Max Dose Reached (%)	Vascular Events (%)	Mortality	Side Effects (%)	Key Findings
Baderkhan et al., 2021 [6]	Sweden	Prospective Cohort	22 months (range 1–98)	40	Started at 100 mg BID; uptitrated every 3–6 months to 400 mg/day	65%	12.5% (5/40) including fatal ruptures	4 deaths from vascular rupture	35% (dizziness, fatigue, headache, etc.)	Pulse/BP reduced; 5 patients stopped due to AEs
Buso et al., 2023 [10]	Italy	Prospective Cohort	72 ± 41 months	26	Up to 400 mg/day; most patients received 400 mg, 1 at 200 mg	61.50%	70% (14/20); 12 vascular events	3 deaths (hepatic, splenic artery rupture)	3.85% (fatigue)	Some events occurred at full dose; mortality noted
Frank et al., 2019 [11]	France	Retrospective Cohort	5.3 years (median)	104	100 mg daily; titrated by 100 mg/month to 400 mg/day	62.50%	26% (27/104)	Improved survival in 400 mg group	3.5% (fatigue)	Hospitalizations and mortality improved at 400 mg
Hoang et al., 2019 [12]	France	Cohort (Abstract)	5 years	43	Not reported	N/A	Not reported	Not reported	Not reported	BP ↑ but changes may reflect aging, not drug effect
Ong et al., 2010 [7]	France and Belgium	RCT (Blinded-Endpoint)	47 ± 5 months	25 (celiprolol)	100 mg increased every 6 months up to 400 mg BID	88%	20% (5/25); control 50% (14/28)	1 death in celiprolol group, 3 in control	12% (mild/moderate fatigue)	RCT: celiprolol reduced vascular events by 64%

## Data Availability

All data sources utilized are available in the Results and Supplementary sections and cited in the references.

## Supplementary Materials

The following supporting information can be downloaded at https://www.mdpi.com/article/10.3390/medsci13020074/s1: Supplementary Table S1. PRISMA checklist; Supplementary Table S2. Risk of Bias assessment of included studies with Newcastle–Ottawa Quality Assessment Scales; Supplementary Table S3. Baseline characteristics of included studies; Supplementary Table S4. Characteristics of included patients; Supplementary Table S5. Celiprolol treatment and its impact; Supplementary Table S6. Celiprolol treatment and side effects.

## Abbreviations

The following abbreviations are used in this manuscript:

ACMG	American College of Medical Genetics
AD	Autosomal Dominant
AGTR1	Angiotensin II Receptor Type 1
BMI	Body Mass Index
BP	Blood Pressure
CI	Confidence Interval
COL1A1	Collagen Type 1 Alpha 1 Chain Gene
COL3A1	Collagen Type III Alpha 1 Chain Gene
DBP	Diastolic Blood Pressure
EDS	Ehlers–Danlos Syndrome
hEDS	Hypermobile Ehlers–Danlos Syndrome
NOS	Newcastle–Ottawa Scale
OR	Odds Ratio
PRISMA	Preferred Reporting Items for Systematic Reviews and Meta-analysis
SBP	Systolic Blood Pressure
TGF-Beta	Transforming Growth Factor Beta
vEDS	Vascular Ehlers–Danlos Syndrome
