# Supplementary material for: The Impact of Celiprolol in Vascular Ehlers–Danlos Syndrome: A Systematic Review of Current Evidence
Source: Med Sci (Basel). 2025 Jun 9;13(2):74. doi: 10.3390/medsci13020074 (PMC12195525; doi:10.3390/medsci13020074)
Supplement: Supplementary file 1 [file medsci-13-00074-s001.zip › medsci-3558294-supplementary.pdf]

**Supplementary Materials**

**Title:** The Impact of Celiprolol in Vascular Ehlers–Danlos Syndrome: A Systematic Review of Current Evidence

**Supplementary Table S1.** PRISMA checklist.

**Supplementary Table S2.** Risk of Bias Assessment of included studies with Newcastle–Ottawa Quality Assessment Scales.

**Supplementary Table S3.** Baseline characteristics of included studies.

**Supplementary Table S4.** Characteristics of included patients.

**Supplementary Table S5.** Celiprolol treatment and its impact.

**Supplementary Table S6.** Celiprolol treatment and side effects.

**Supplementary Table S1.** PRISMA checklist.

## PRISMA 2020 Checklist

| Section and Topic | Item # | Checklist item | Location where item is reported |
|---|---|---|---|
| **TITLE** | | | |
| Title | 1 | Identify the report as a systematic review. | 1 |
| **ABSTRACT** | | | |
| Abstract | 2 | See the PRISMA 2020 for Abstracts checklist. | 1 |
| **INTRODUCTION** | | | |
| Rationale | 3 | Describe the rationale for the review in the context of existing knowledge. | 2 |
| Objectives | 4 | Provide an explicit statement of the objective(s) or question(s) the review addresses. | 2 |
| **METHODS** | | | |
| Eligibility criteria | 5 | Specify the inclusion and exclusion criteria for the review and how studies were grouped for the syntheses. | 3 |
| Information sources | 6 | Specify all databases, registers, websites, organisations, reference lists and other sources searched or consulted to identify studies. Specify the date when each source was last searched or consulted. | 3 |
| Search strategy | 7 | Present the full search strategies for all databases, registers and websites, including any filters and limits used. | 3 |
| Selection process | 8 | Specify the methods used to decide whether a study met the inclusion criteria of the review, including how many reviewers screened each record and each report retrieved, whether they worked independently, and if applicable, details of automation tools used in the process. | 3 |
| Data collection process | 9 | Specify the methods used to collect data from reports, including how many reviewers collected data from each report, whether they worked independently, any processes for obtaining or confirming data from study investigators, and if applicable, details of automation tools used in the process. | 3 |
| Data items | 10a | List and define all outcomes for which data were sought. Specify whether all results that were compatible with each outcome domain in each study were sought (e.g. for all measures, time points, analyses), and if not, the methods used to decide which results to collect. | 3 |
| | 10b | List and define all other variables for which data were sought (e.g. participant and intervention characteristics, funding sources). Describe any assumptions made about any missing or unclear information. | 3 |
| Study risk of bias assessment | 11 | Specify the methods used to assess risk of bias in the included studies, including details of the tool(s) used, how many reviewers assessed each study and whether they worked independently, and if applicable, details of automation tools used in the process. | 3 |
| Effect measures | 12 | Specify for each outcome the effect measure(s) (e.g. risk ratio, mean difference) used in the synthesis or presentation of results. | 3 |
| Synthesis methods | 13a | Describe the processes used to decide which studies were eligible for each synthesis (e.g. tabulating the study intervention characteristics and comparing against the planned groups for each synthesis (item #5)). | 3 |
| | 13b | Describe any methods required to prepare the data for presentation or synthesis, such as handling of missing summary statistics, or data conversions. | 3 |
| | 13c | Describe any methods used to tabulate or visually display results of individual studies and syntheses. | 3 |
| | 13d | Describe any methods used to synthesize results and provide a rationale for the choice(s). If meta-analysis was performed, describe the model(s), method(s) to identify the presence and extent of statistical heterogeneity, and software package(s) used. | 3 |
| | 13e | Describe any methods used to explore possible causes of heterogeneity among study results (e.g. subgroup analysis, meta-regression). | 3 |
| | 13f | Describe any sensitivity analyses conducted to assess robustness of the synthesized results. | Supplementary |
| Reporting bias assessment | 14 | Describe any methods used to assess risk of bias due to missing results in a synthesis (arising from reporting biases). | Supplementary |
| Certainty assessment | 15 | Describe any methods used to assess certainty (or confidence) in the body of evidence for an outcome. | 3 |
| **RESULTS** | | | |
| Study selection | 16a | Describe the results of the search and selection process, from the number of records identified in the search to the number of studies included in the review, ideally using a flow diagram. | 4 |

## PRISMA 2020 Checklist

| Section and Topic | Item # | Checklist item | Location where item is reported |
|---|---|---|---|
| **RESULTS** | | | |
| Study selection | 16a | Describe the results of the search and selection process, from the number of records identified in the search to the number of studies included in the review, ideally using a flow diagram. | 4 |
| | 16b | Cite studies that might appear to meet the inclusion criteria, but which were excluded, and explain why they were excluded. | 5 |
| Study characteristics | 17 | Cite each included study and present its characteristics. | 5 |
| Risk of bias in studies | 18 | Present assessments of risk of bias for each included study. | Supplementary |
| Results of individual studies | 19 | For all outcomes, present, for each study: (a) summary statistics for each group (where appropriate) and (b) an effect estimates and its precision (e.g. confidence/credible interval), ideally using structured tables or plots. | 5-11 |
| Results of syntheses | 20a | For each synthesis, briefly summarise the characteristics and risk of bias among contributing studies. | 5-6 |
| | 20b | Present results of all statistical syntheses conducted. If meta-analysis was done, present for each the summary estimate and its precision (e.g. confidence/credible interval) and measures of statistical heterogeneity. If comparing groups, describe the direction of the effect. | N/A |
| | 20c | Present results of all investigations of possible causes of heterogeneity among study results. | N/A |
| | 20d | Present results of all sensitivity analyses conducted to assess the robustness of the synthesized results. | N/A |
| Reporting biases | 21 | Present assessments of risk of bias due to missing results (arising from reporting biases) for each synthesis assessed. | 5-6 |
| Certainty of evidence | 22 | Present assessments of certainty (or confidence) in the body of evidence for each outcome assessed. | 5-11 |
| **DISCUSSION** | | | |
| Discussion | 23a | Provide a general interpretation of the results in the context of other evidence. | 5-6 |
| | 23b | Discuss any limitations of the evidence included in the review. | 12 |
| | 23c | Discuss any limitations of the review processes used. | 12 |
| | 23d | Discuss implications of the results for practice, policy, and future research. | 11-12 |
| **OTHER INFORMATION** | | | |
| Registration and protocol | 24a | Provide registration information for the review, including register name and registration number, or state that the review was not registered. | 2 |
| | 24b | Indicate where the review protocol can be accessed, or state that a protocol was not prepared. | 4-6 |
| | 24c | Describe and explain any amendments to information provided at registration or in the protocol. | N/A |
| Support | 25 | Describe sources of financial or non-financial support for the review, and the role of the funders or sponsors in the review. | 9-11 |
| Competing interests | 26 | Declare any competing interests of review authors. | 11 |
| Availability of data, code and other materials | 27 | Report which of the following are publicly available and where they can be found: template data collection forms; data extracted from included studies; data used for all analyses; analytic code; any other materials used in the review. | 11-12 |

**Supplementary Table S2.** Risk of Bias Assessment of included studies with Newcastle–Ottawa Quality Assessment Scales.

| Author, Year | Study | Selection (0-4) | Comparability (0-2) | Outcome (0-3) | Total | Category |
|---|---|---|---|---|---|---|
| Baderkhan et al., 2021 | Cohort study | 4 | 2 | 3 | 9 | Low Risk |
| Buso et al., 2023 | Cohort study | 3 | 2 | 3 | 8 | Low Risk |
| Frank et al., 2019 | Cohort study | 4 | 2 | 3 | 9 | Low Risk |
| Hoang et al., 2014 | Cohort study | 3 | 2 | 3 | 8 | Low Risk |
| Ong et al, 2010 | Cohort study | 4 | 2 | 3 | 9 | Low Risk |

**Supplementary Table S3.** Baseline characteristics of included studies.

| No | Author | Study Period | Follow-up Period | Study Design | Country | Sample Size | Age | Man n (%) | BMI |
|---|---|---|---|---|---|---|---|---|---|
| 1 | Baderkhan et al., 2021 | 2011-2019 | 22 (1-98 months) | Prospective Cohort | Sweden | 40 | 43.5 (15±78) | N/A | N/A |
| 2 | Buso et al., 2023 | 2011-2023 | 72 ± 41 months | Prospective Cohort | Italy | 26 | 37 (16) | 10 (38%) | 21.6 ± 3.1 |

| | | | | | | | | | |
|---|---|---|---|---|---|---|---|---|---|
| 3 | Frank et al., 2019 | 2000-2017 | 5.3 (3.2–8.5) years | Retrospective Cohort | France | -Celiprolol only: 104<br>- Celiprolol and other drugs: 26<br>- Other drugs: 8<br>- No treatments: 6 | 34.5 (25.0–42.5) | 57 (39.6%) | 21.2 (19.0–23.7) |
| 4 | Hoang et al., 2019 | N/A | 5 years | Cohort | France | - (+) Celiprolol: 43 (73%)<br>- (-) Celiprolol: 17 (27%) | 35 ± 10 | 27 (43%) | N/A |
| 5 | Ong et al, 2010 | 2003-2006 | 47 ± 5 months | Retrospective Cohort | France and Belgium | Celiprolol: 25<br>Control: 28 | Celiprolol: 36 ± 13<br>Control: 35 ± 11 | Celiprolol: 8 (32%)<br>Control: 10 (40%) | Celiprolol: 20 ± 2<br>Control: 21 ± 4 |

**Supplementary Table S4.** Characteristics of included patients.

| No | Author | Comorbidities | | | | | | EDS type of variants |
|---|---|---|---|---|---|---|---|---|
| | | DM (%) | HT (%) | Dyslipidemia (%) | Past Arterial events | Past GI events | Past Pulmonary Events | |
| 1 | Baderkhan et al., 2021 | 2 (5%) | 27 (67.5%) | N/A | 20 (50%) | 5 (12%) | 3 (7%) | **American College of Medical Genetics (ACMG) interpretation**<br>- 3: 1<br>- 4: 10<br>- 5: 9 |
| 2 | Buso et al., 2023 | 5 (19%) | 8 (31%) | 2 (12%) | 7 (27%) | 4 (15%) | N/A | - Glycine substitutions in the triple-helical domain of COL3A1.: 13 (50%)<br>- Splice site mutations: 4 (15.4%)<br>- Novel glycine substitution mutation: 1 (3.8%)<br>- Deletion/insertion mutation likely resulting in haploinsufficiency: 1 |

| | | | | | | | | |
|---|---|---|---|---|---|---|---|---|
| | | | | | | | | (3.8%) |
| 3 | Frank et al., 2019 | N/A | N/A | N/A | 74 (51.4%) | 33 (22.9%) | 15 (10.4%) | Group I (glycine missense): 90 (62.5%)<br>Group II (splice-site variants, insertions-deletions, duplications.): 42 (29.2%)<br>Group III (variants leading to haploinsufficiency.): 12 (8.3%) |
| 4 | Hoang et al., 2019 | N/A | N/A | N/A | N/A | N/A | N/A | N/A |
| 5 | Ong et al, 2010 | N/A | N/A | N/A | Only personal history of arterial rupture or dissection, uterine or intestinal rupture<br><br>Celiprolol: 14 (56%)<br>Control: 15 (53.6%) | | | Genotype:<br>**COL3A1 mutation**<br>Celiprolol: 13 (54.2%)<br>Control: 20 (83.3%) |

**Supplementary Table S5.** Celiprolol treatment and its impact.

| No | Author, Year | Doses | Doses at the end of follow up | Mean time from baseline visit to maximum recommended dose | Side Effects | Vascular events during treatment | GI events during treatment | Pulmonary events during treatment | Mortality [Rationale] | Other reported outcomes |
|---|---|---|---|---|---|---|---|---|---|---|
| 1 | Baderkhan et al., 2021 | - Started with 100mg twice daily, uptitrated every 6 months.<br>- If well tolerated, changed to every 3 months) to 400mg/day. | - 100mg: 4 (10%)<br>- 200mg: 5 (12.5%)<br>400mg in 26 (65%)<br>- Treatment termination: 5 (12.5%) | N/A | - Dizziness: 5 (12.5%)<br>- Abnormal tiredness: 4 (10%)<br>- Headache: 2 (5%)<br>- Nausea: 2 (5%)<br>- Tendency to fall: 1 (2.5%)<br>- Diplopia: 1 (2.5%)<br>- Bradycardia: 1 (2.5%)<br>- Arthralgia : 1 (2.5%)<br>- Syncope: 1 (2.5%)<br>- Paraesthesia | - Type A aortic dissection: 1 (2.5%)<br>- Cerebral aneurysm: 1 (2.5%)<br>- Ascending aortic rupture: 1 (2.5%)<br>- Pulmonary artery rupture: 1 (2.5%)<br>- Internal iliac artery bleeding: 1 (2.5%) | N/A | N/A | 4 patients (Type A aortic dissection (n=1; 2.5%); Subarachnoid bleeding (n=1; 2.5%); Ascending aortic rupture (n=1; 2.5%) ; Pulmonary artery rupture (n=1; 2.5%)] | - Mean pulse rate changes: 76 ± 5.7 to 70 ± 8.1 bpm (p= .14).<br>- Mean systolic BP changes: 127 ± 14.5 to 120 ± 13.3 mmHg.<br>- Mean diastolic BP changes: 82 ± 14.2 to 75 ± 11.5 mmHg.<br>- Mean pulse pressure changes: 45 ± 10.8 to 44 ± 14.2 mmHg. |

| | | | | | | | | | | | |
|---|---|---|---|---|---|---|---|---|---|---|---|
| | | | | | of the fingers: 1 (2.5%)<br>- Anxiety and sexual dysfunction: 1 (2.5%) | | | | | | |
| 2 | Buso et al., 2023 | N/A | - 400 mg daily: 16 (61.54%)<br>- 200 mg daily: 1 (3.85%) | 32 ± 30 months | Only one patient (3.85%) had to stop taking celiprolol because of fatigue. | -Type B aortic dissection: 2 (7.69%)<br>- Renal artery thrombosis or dissection with renal infarction: 3 (11.54%)<br>- Hepatic artery rupture: 2 (7.69%)<br>- Splenic artery rupture (3.85%)<br>- Iliac artery thrombosis or dissection: | - Spontaneous sigmoid colon perforation: 1 (3.85%)<br>- Appendicular perforation: 1 (3.85%)<br>- Spontaneous spleen rupture: 2 (7.69%)<br>- Gallbladder rupture: 1(3.85%) | Spontaneous hemothorax: 1 (3.85%) | 3 patients [Type B aortic dissection and rupture of hepatic arteries during splenectomy (n=2; 7.69%) Splenic artery rupture (n=1; 3.85%)] | N/A | |

| | | | | | | | | | | |
|---|---|---|---|---|---|---|---|---|---|---|
| | | | | | | 3 (11.54%)<br>- Spontaneous hematoma of the iliopsoas or thigh: 2 (7.69%) | | | | |
| 3 | Frank et al., 2019 | - Initial dosing of celiprolol was 100 mg once daily.<br>- Increased by 100 mg/day every month over a 3-month period to reach a maximum dose of 400 mg/day (200 mg twice daily). | 400 mg/day: 90 (62.5%)<br>Required dose reduction: 5 (3.5%) | N/A | - Fatigue: 5 (3.5%) | Celiprolol: 27 (26.0%)<br>Celiprolol + Other drugs: 13 (50.0%) | Celiprolol: 9 (8.6%)<br>Celiprolol + Other drugs: 2 (7.7%) | Celiprolol: 0 (9.6%)<br>Celiprolol + Other drugs: 1 (3.8%) | Group I: 13 (76.5%)<br>Group II: 4 (23.5%)<br>Group III: 0 (0%) | - The observed reduction in mortality was dose-dependent.<br>- There is a statistically significant difference in the ratio of hospitalizations for acute arterial events and regular follow-up. |
| 4 | Hoang et al., 2019 | N/A | N/A | N/A | N/A | N/A | N/A | N/A | N/A | - SBP increased with time under celiprolol (0.79 |

| | | | | | | | | | | |
|---|---|---|---|---|---|---|---|---|---|---|
| | | | | | | | | | | mmHg/y, p<0.001), so did central SBP (0.89 mmHg/y; pZ0.002) and central PP (1.24 mmHg/y, p<0.001), without changed heart rate.<br>- Di and IMT increased (+36mm/y, p<0.001 and +4.4 mm/y, p<0.001, respectively). Einc increased (29.92 kPa/y, p<0.001) and distensibility decreased (-0.003 kPa-1/y, p<0.001).<br>- In unexposed |

| | | | | | | | | | | |
|---|---|---|---|---|---|---|---|---|---|---|
| | | | | | | | | | | patients (nZ17), brachial BP did not change significantly, whereas changes in arterial wall properties were similar to those exposed to celiprolol.<br><br>(Carotid internal diastolic diameter (Di), intima-media thickness (IMT), arterial wall cross-sectional (WCSA), circumferential wall stress, distensibility, and Young’s |

| | | | | | | | | | | |
|---|---|---|---|---|---|---|---|---|---|---|
| | | | | | | | | | | elastic modulus (Einc)) |
| 5 | Ong et al, 2010 | - Celiprolol was uptitrated 100 mg every 6 months to a maximum of 400 mg twice daily. | Target dose of 400 mg twice a day was reached in all but two patients | N/A | N/A | N/A | N/A | N/A | N/A | - The primary endpoints of arterial events were reached by five (20%) in the celiprolol group and by 14 (50%) controls<br>- Adverse events were severe fatigue in one patient after starting 100 mg celiprolol and mild fatigue in two patients related to dose up titration.<br>- Treatment of patients with |

| | | | | | | | | | | |
|---|---|---|---|---|---|---|---|---|---|---|
| | | | | | | | | | | celiprolol compared with no treatment reduced arterial events, such as rupture or dissection, by three times and was effective even after adjustment for genotype. |

**Supplementary Table S6.** Celiprolol treatment and side effects.

| No | Author, Year | Initial Dose | Target Dose (Uptitration) | Participants reaching maximum recommended dose (%) and End- | Participants experiencing Side Effects (%) | Side effects | Severe side effects | Rationale of not reaching maximum recommended doses | Rationale of improving side effects | Rationale of stopping Celiprolol |
|---|---|---|---|---|---|---|---|---|---|---|

| | | | | | | | | | | |
|---|---|---|---|---|---|---|---|---|---|---|
| | | | | **dose detail** | | | | | | |
| 1 | Baderkhan et al., 2021 | 100mg | 400mg twice daily (Uptitrated every 6 months) | 65%<br><br>- 100mg: 4 (10%)<br>- 200mg: 5 (12.5%)<br>400mg in 26 (65%)<br>- Treatment termination: 5 (12.5%) | 35% | - Dizziness: 5 (12.5%)<br>- Abnormal tiredness: 4 (10%)<br>- Headache: 2 (5%)<br>- Nausea: 2 (5%)<br>- Tendency to fall: 1 (2.5%)<br>- Diplopia: 1 (2.5%)<br>- Bradycardia: 1 (2.5%)<br>- Arthralgia : 1 (2.5%)<br>- Syncope: 1 (2.5%)<br>- Paraesthesia of the fingers: 1 (2.5%)<br>- Anxiety and sexual | 15% (6/40) | Headache: 1 (2.5%)<br><br>Imbalance: 1 (2.5%)<br><br>Dizziness: 2 (5%)<br><br>Hypotension: 1 (2.5%)<br><br>Deterioration of asthma: 1 (2.5%) | N/A | Severe side effects: 4 (10%)<br><br>Alleged economic reasons: 1 (2.5%) |

| | | | | | | | | | | |
|---|---|---|---|---|---|---|---|---|---|---|
| | | | | | | dysfunction: 1 (2.5%) | | | | |
| 2 | Buso et al., 2023 | N/A | 400mg daily | 61.54%<br><br>- 400 mg daily: 16 (61.54%)<br>- 200 mg daily: 1 (3.85%) | N/A | N/A | 1 (3.85%) | Fatigue: 1 (3.85%) | N/A | N/A |
| 3 | Frank et al., 2019 | 100mg once daily | 400mg/day ,200mg twice daily | 62.5%<br><br>400 mg/day: 90 (62.5%)<br>Required dose reduction: 5 (3.5%) | 3.5% | N/A | Fatigue: 5 (3.5%) | Fatigue: 5 (3.5%) | N/A | N/A |
| 4 | Hoang et al., 2019 | N/A | N/A | N/A | N/A | N/A | N/A | N/A | N/A | N/A |

| | | | | | | | | | | |
|---|---|---|---|---|---|---|---|---|---|---|
| 5 | Ong et al, 2010 | 100 mg | 400 mg twice daily (100mg every 6 month) | 88% | 12% | Mild Fatigue: 2 (8%) | 1/25 (4%) | Severe Fatigue: 1 (4%) | N/A | Severe fatigue: 1 (4%) |
